# Perceived Challenges and Online Harms from Social Media Use on a Severity Continuum: A Qualitative Psychological Stakeholder Perspective

**DOI:** 10.3390/ijerph18063227

**Published:** 2021-03-20

**Authors:** Melina A. Throuvala, Mark D. Griffiths, Mike Rennoldson, Daria J. Kuss

**Affiliations:** 1 International Gaming Research Unit, Psychology Department, Nottingham Trent University, Nottingham NG1 4FQ, UK; mark.griffiths@ntu.ac.uk (M.D.G.); daria.kuss@ntu.ac.uk (D.J.K.); 2 Psychology Department, Nottingham Trent University, Nottingham NG1 4FQ, UK; mike.rennoldson@ntu.ac.uk

**Keywords:** social media, online challenges, online harms, school prevention, adolescence, social media impacts

## Abstract

Evidence suggests that problematic use of gaming, the internet, and social media among adolescents is on the rise, affecting multiple psycho-emotional domains. However, research providing a comprehensive and triangulated stakeholder perspective of perceived harms is lacking. How are adolescent online harms experienced and conceptualized by students, parents, and teachers? The present study comprised part of a qualitative needs assessment investigation with the use of focus groups and individual interviews among key stakeholder groups assessing perceived impacts with a focus on the negative consequences and perceived harms. The study’s sample consisted of students (*N* = 42, *M_age_* = 13.5, *SD* = 2.3), parents (*N* = 9, *M_age_* = 37, *SD* = 5.6) and teachers (*N* = 9, *M_age_ =* 34, *SD* = 4.9) from the UK. Data were analysed with thematic analysis. Findings focused primarily on social media use impacts and indicated that processes underlying impacts experienced by adolescents may be conceptualized on a severity continuum. Stakeholder consensus on perceptions of challenges and perceived harms formed the second theme, with impacts further analysed as relating to time displacement, peer judgement, sensory overload and context of the adolescent with functional (performance, task switching, use of multiple devices), cognitive (loss or deterioration of attentional focus, attention deficit), and emotional consequences (stress, anxiety, obsessive-compulsive/checking behaviours). A third theme formed was individual vulnerabilities predisposing poor mental health outcomes. The final theme related to impacts dependent on context and meaning attached. Findings suggest a consideration of a spectrum approach encompassing a broader range of potential psychological challenges and perceived harms beyond safety concerns and addiction in understanding problematic adolescent online experiences. Understanding perceived harms can aid the objective setting of interventions and consideration of mental health literacy in school curricula.

## 1. Introduction

Children and adolescents are increasingly engaged in technologies primarily to gather social capital, maintain constructive interpersonal relationships, and seek help online [1]. Time spent on non-digital interactions between 2003 and 2017 for adolescents and young adults in the US has demonstrated declines of 140 h a year [2], which suggests an increase in digital use in young people. However, excessive use of digital activities and smartphones may result in multiple mental and physical problems, such as behavioural addiction, cognitive impairment, and emotional distress [1,3]. More specifically, in the UK, a sharp rise in children and adolescents in the UK being treated by National Health Service (NHS) mental health services has been observed (from 9.7% in 1999 to 12.8% in 2017—including those aged up to 19 years) with emotional disorders rising from 4.3% in 1999 to 5.8% in 2017 and one in six (16.9%) aged 17 to 19 years having a mental disorder [4].

This rise in mental health needs is fuelled, in addition to large socioeconomic and individual factors, by exposure and higher prevalence to social media [5], which is associated with the development of problematic use among adolescents [6]. Problematic social media use presents with cognitive, emotional, and behavioural symptoms, which manifest as harms in the form of negative consequences in an individual’s life [7]. This should be distinguished from addictive use—which includes symptoms of preoccupation, tolerance, withdrawal, loss of control, conflict and relapse [8]. Confusion between problematic use and addictive use may have resulted in uncertainty about prevalence rates. A recent meta-analysis of social media addiction prevalence across 32 nations identified variations depending on classification and cut-off points, ranging from 5% to 25% [9].

Despite social media addiction not being a legitimate clinical entity and, to date, a debatable psychological construct [10], there are conceptual similarities with problematic media use [11]. Impacts from social media use are compounded by the constant accessibility utilizing smartphones, which has fuelled a proliferation of research on smartphone use [12]. A systematic literature review and meta-analysis involving data from 41,000 individuals indicated a high level of problematic smartphone use (PSU) (one in four children and adolescents) and an association with mental health symptoms (depression, anxiety, high levels of perceived stress, and poor sleep) [13]. However, despite evidence supporting that the more time spent online increases the possibility of exposure to risks and pathological tendencies (e.g., cyberbullying) and potential benefits (e.g., enhancing social relationships), longitudinal evidence that time spent using social media is detrimental for mental health over time is still conflicting and highly debated [14,15,16,17,18,19,20,21], with academic debates arguing the thresholds of habitual, problematic, and addictive use [22] associated with psychological harm [23].

Psychological harm can be defined as impacts on an individual’s well-being and psyche [24]. The literature has identified a wide variety of psychosocial harms and impacts associated with excessive social media and smartphone use which can arise beyond use [25]: poor academic performance and classroom hostility in adolescents [26,27], sleep, Attention Deficit Hyperactivity Disorder (ADHD) and family problems cyberbullying, interpersonal relationship difficulties, psychological problems (i.e., anxiety, depression) and distress [28,29,30,31,32,33,34], negative self-perception, negative interactions, aggressive acts and exposure to harmful content (promoting self-harm or suicidality), arguably in a dose–response relationship [23]. Increased screen time has been suggested as a factor contributing to shorter sleep duration among adolescents [35]. Social media use may be conferring subtle changes on cognitive capacity, such as retention of information and recall [36] and there is an emergent body of literature on neurophysiological changes [37]. Cognitive impacts have also been suggested, i.e., distraction from smartphone use on daily functioning and academic performance) and constitute an emerging area of research [38,39]. Both adolescents (54%) and 72% of parents have reported that mobile devices are a daily source of distraction for adolescents, while parents are being reported as similarly distracted by devices daily (50% of parents and 44% of adolescents) [40]. However, impacts have been reported to be beyond a binary conceptualization presenting with both positive and negative effects (social interactions may promote closeness and distance; self-expression may promote growth and social comparison [41,42,43,44,45,46]).

Evidence regarding problematic internet use among adult populations refers to many different activities (i.e., general surfing, online shopping, use of online auction websites, social networking, use of online pornography, etc.) with age being a significant moderator for the majority of these activities [47]. In the context of adolescence, PIU primarily refers to social media and gaming activities with evidence for other problematic activities, such as compulsive streaming (e.g., watching YouTube videos) [48] or exposure to online pornography [49]. The majority of the previous literature has focused on time spent on social media and/or gaming as evidence for problematic use, and as an increasingly prevalent aspect in sedentary behaviours overshadowing other more traditional behaviours (e.g., television viewing) [50]. However, despite evidence supporting that the more time spent online increases the possibility of exposure to risks and pathological tendencies (e.g., cyberbullying) and potential benefits (e.g., enhancing social relationships), longitudinal evidence that time spent using social media is detrimental for mental health over time is still conflicting and highly debated [14,15,16,17,18,19,20,21], with academic debates arguing the thresholds of normative and problematic behaviours.

Recent evidence has expanded the scope of inquiry into specific behaviours associated with problematic outcomes beyond time spent engaged in the activity: compulsive texting and diminishing academic performance [51]; daily interruptions and reduced productivity [52]; excessive selfie-taking and negative comparisons associated with narcissistic tendencies, disordered eating, body image concerns and body dysmorphic disorder [53,54,55]; the experience of fear of missing out (FoMO; missing out on pleasurable activities), and nomophobia (‘no mobile phone’ phobia) [56,57]; compulsive use and checking behaviours [58,59]; exhibiting aggression, cyberbullying and cyberstalking [60]; phubbing (snubbing an individual by using ones smartphone instead of engaging socially) [61]; sexting and sext-sharing [62]; and problematic smartphone use [63,64].

Increasing concerns over online harms have given rise to a number of governmental and non-governmental bodies’ responses. For example, in the UK, concerns over children’s vulnerability to harmful content [65] iterated initially in the “Internet Safety Strategy Green Paper” [66] led to a governmental response for an open call for evidence and recommendations for a new regulatory framework to inform online safety regulation and address the negative impacts of social media and screen time on young people [67,68]. This process then led to: (i) the development of an “Online Harms White Paper” [69] outlining an extended range of online harms and legislative and non-legislative ways to address these amongst social media operators, schools, parents and carers, and other stakeholders, and (ii) the development of an age-appropriate design code of practice [70]. The latter report outlined a code of practice for providers of online social media platforms as a guideline and a general approach to age-appropriate content, defining social media operators’ duty for robust provision of age verification systems and age-appropriate services and ways operators can involve and support parental involvement in the process [71]. Ofcom, the UK’s telecommunications’ regulator, was also given interim power to regulate the activity of social media operators (i.e., to impose fines or even suspend operations) if they failed to comply with legislation regarding harmful content online (i.e., violence or child abuse) [72] similar to scholarly initiatives for gambling harm reduction [73,74].

These governmental approaches for stronger intervention have been complemented by initiatives from charities and non-profit organizations to support educators, schools, and other stakeholders on media literacy efforts with a governmental intention to coordinate all these activities, assess areas of duplication and overlap, and coordinate a country-wide media literacy strategy and an overarching statutory duty of care [68]. The UK Government recently announced the introduction of the Online Safety Bill as a final response to the Online Harms White Paper, initiating a new regulatory framework to target illegal or harmful online content and greater legal accountability on online companies [75]. Similar approaches have been emerging in other European countries [76] with East Asian countries leading more externally regulated restrictive approaches [77,78,79] due to the high prevalence rates in problematic online use [80].

Despite the notable impacts highlighted in the extant literature, children and young people’s voices are often overlooked in terms of how these impacts are conceptualized and how proposed changes regarding recommendations are implemented [81]. Additionally, teacher and parent accounts have been independently assessed [82,83], but no multiple stakeholder accounts exist to provide consensus on online challenges experienced during adolescence [84]. Adolescence is a developmental period with high vulnerability to mental illness [85] and most mental health disorders have their onset during adolescence [86]. Additionally, this period is one of the most critical times since adolescents are laying down the foundations for their academic and professional choices, while it is a period of risk-taking behaviours with difficulties in emotion regulation ability. Risk-taking behaviours may be used as a coping mechanism and contribute to poor mental health jeopardizing adolescents’ mental and physical well-being [87]. Risky behaviours and poor mental health initiating in childhood can define future development [13]. Recent findings using objective *Facebook* data have confirmed a direct association between frequency and intensity of positive feedback in the form of likes with perceived well-being [88]. Adolescents, who experience a higher vulnerability to peer evaluations, may similarly experience the rewarding aspects of social media, but may also present with emotion regulation needs [89] or overreliance on excessive reassurance-seeking behaviours which could be the gateway to PSU [90,91]. Therefore, understanding challenges and perceived harms in adolescence may facilitate prevention measures to address expectancies and cognitions related to rewards and enhance skill development and healthy coping at a school level [92,93].

To better understand the needs and concerns of stakeholders, it is necessary to develop an understanding of current perceived harms experienced by adolescents in relation to their online use. It is important to understand outcomes as these may predict problematic trajectories [94]. The present study attempts to provide a conceptual taxonomy of online challenges and perceived harms for adolescents arising from social media experiences to facilitate the development of an assessment of the nature of psychological harm-related issues beyond safety concerns, adding to the growing evidence base. Understanding stakeholder conceptualizations—student, parent, and teacher—for online-related harm for adolescents may aid in developing a more coherent understanding of harms for research, policy formation, and treatment provision for mental health [95].

## 2. Materials and Methods

### 2.1. Design

The present study utilised thematic analysis (TA) to analyse the data collected [96]. TA is among the most widely used methods in conducting qualitative analysis [97] and is a theoretically flexible qualitative method used to identify, analyse and interpret patterns of meaning leading to the development of key common themes [96]. It is a useful method to explore different participant perspectives or to decipher newly emergent issues and may provide rich and complex insights [98]. A constructionist viewpoint was employed, which argues for meaning that is socially determined and co-produced [99]. Emphasis was placed on the perceived challenges and perceived harms of online engagement.

Parent and teacher perspectives on perceived adolescent challenges and harms were also provided as part of a larger stakeholder study to inform key recommendations for media literacy education priorities. For the purposes of the present study, triangulation involved the recruitment of multiple types of key informants (i.e., adolescent students, parents, and teachers) to explore concerns from different perspectives [100]. Employing multiple evaluation strategies to allow for triangulation of data [100,101] has been recommended as an optimal approach to assess effectiveness of interventions [102]. Triangulation, which involves a combination of different methods or data sources as a way to validate results, increases the scope and the depth of research findings [103], strengthens the external validity of the study [104], and reinforces the trustworthiness of data [105]. External validity is defined by the degree of generalisability of the findings to other studies independent of population used, timing or settings [106].

### 2.2. Participants

Three different stakeholder groups were used in the present study, which comprised of six student focus groups and 18 parent and teacher interviews to obtain consensus on concerns and challenges experienced. Stakeholder approaches provide a more comprehensive account of adverse online experiences, which may aid harm reduction and intervention [107]. Triangulation of data sources was sought to explore commonalities and differences in the conceptualisation of harms [108]. Therefore, multiple informants were recruited (students, parents, and teachers) so the construct of challenges and perceived harms could be explored from different perspectives [84,109], allowing exploration across different groups to reinforce the trustworthiness of data [105]. Focus groups were conducted with the students to facilitate discussion and elicit different viewpoints and experiences based on the semi-structured guide, followed by interviews with parents and teachers for an in-depth understanding of their perceptions and meaning making on the topic [110]. The sociodemographic characteristics of the three groups are outlined below.

#### 2.2.1. Students

Participants (*N* = 42) aged 12–16 years (*M* = 13.5 years, *SD* = 2.3) were sampled in collaboration with three local secondary schools in the East Midlands area of the UK, including a mix of an all-female school and two co-educational schools. Students were primarily white (63%), black (22%) and Asian (15%), with an almost even gender split (48% female). The study targeted adolescents due to the: (i) high online usage this age group exhibits, and the vulnerability to peer evaluations and risk behaviours [111]; (ii) heightened vulnerability to excessive online use, potentially leading to addictive symptoms [112]; and (iii) development of body-image concerns and an overemphasis on peer comparisons that may be associated with the development of eating disorders, obesity, and dysfunctional exercise [113,114].

#### 2.2.2. Parents

Participants (*N* = 9), aged 39–53 years (*M*_age_ = 44.78, *SD* = 5.04), were parents of adolescent children selected in collaboration with three local secondary schools in the East Midlands area of the UK, including a mix of an all-female school and two co-educational schools. Participants were primarily white (*n* = 5), black (*n* = 3) and Asian (*n* = 1), comprising six females and three males, and from diverse socioeconomic communities: upper socioeconomic group (*n* = 4), middle (*n* = 4), and lower (*n* = 1). The study targeted parents due to: (i) the need to identify parental concerns as a critical source of input regarding adolescent problems arising from use; (ii) a lack of studies reflecting the parental perspective of intervention needs for adolescents in relation to problems from online use; and (iii) adolescents being a critical cohort due to their developmental stage, which presents with vulnerable online behaviours and a major influence from peers [111,115]; and (iv) a growing need for family-based prevention strategies [116].

#### 2.2.3. Teachers

Participants (*N* = 9), aged 29–52 years (*M*_age_ = 39.2, *SD* = 7.74), were teachers in UK secondary education (Year 8–12) of three local schools in the East Midlands area of the UK, including a mix of an all-female school and two co-educational schools. Participants were primarily white (*n* = 7), black (*n* = 1) and Asian (*n* = 1), comprising five females and four males, and from middle (*n* = 5), and lower (*n* = 4) socioeconomic backgrounds. The study targeted teachers due to: (i) the need to identify teacher perspectives and concerns regarding adolescent online problems; (ii) a lack of studies reflecting teacher views for prevention purposes [117]; (iii) evidence of higher efficacy of intervention effects when greater teacher commitment was displayed [118]; and (iv) a growing need for school-based prevention strategies [92].

### 2.3. Procedure

Ethical approval for the study was granted by the research team’s university Ethics Committee (No. 2017/109). Focus group discussions with students and parent and teacher interviews focused on a semi-structured guide, which was developed to elicit key perceptions of challenges and perceived psychological harms from the use of social media experienced during adolescence from a multiple stakeholder perspective. Data were collected from six adolescent focus groups, nine teachers, and nine parents with children from three schools in the East Midlands area over a period of two months. Upon agreement for participation, information sheets about the nature of the study were distributed electronically from the school administration to the parental community, along with parental opt-out forms. Teachers were designated by the schools upon the school’s invitation for participation in the present research study. The sign-up for participation was therefore conducted by the schools’ administrators and there was no compensation or remuneration provided for participation. Each student focus group and parent and teacher interview lasted 60 to 100 min, and was audio-recorded and transcribed verbatim with the use of *NVivo 12.00* software. Adolescents were asked to discuss perceptions of challenges and perceived psychological harms personally experienced and/or observed among their peers in relation to internet use and their main activities. However, organically and uniformly, the discussion both in the focus groups and the interviews focused on social media use. The study was part of a larger exploration in relation to specific uses and practices, motivations for use, problems and concerns to adolescent online engagement to inform intervention needs.

### 2.4. Data Analysis

Braun and Clarke [96] proposed six steps of TA to be followed: (i) familiarizing oneself with the data by transcribing, reviewing, and annotating the data to reflect on initial ideas, (ii) generating initial codes, (iii) searching for broader level themes by sorting and grouping the codes, (iv) reviewing themes by cutting, collapsing, or breaking apart initial themes, (v) defining and naming themes by capturing the essence of what each theme is about, and (vi) producing the report with extracts embedded in an analytic narrative. Coding may involve transcription by different authors to support data analyses and triangulation [119]. Coding was implemented by two members of the research team to allow for a reflexive approach and an active process of interpretation and engagement with the analytical process [120]. Where discrepancies arose, these were discussed until consensus was reached. Participant identifiers were constructed by the number of the focus group or interview, the gender, and the participant number which corresponded to the selected quote (i.e., “FG1” means the first focus group), followed by a reference to gender (M = male, F = female) and participant number (i.e., F5). Consequently, the code “FG1F5” refers to a quote from female participant number five in the first focus group. Similarly, participants in interviews were designated by reference to interview (I = Interview), the number assigned to the participant, the gender of the participant (i.e., I6F) followed by the age and the function of the participant (parent or teacher).

## 3. Results

### 3.1. Stakeholders’ Perceptions of Impacts

**Figure 1:**
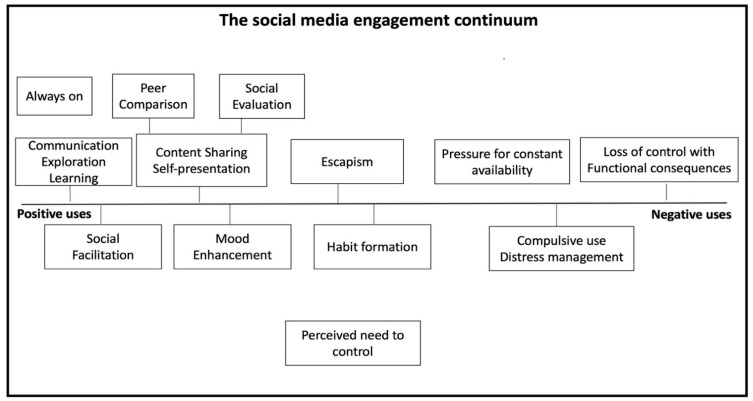
Continuum of perceived processes experienced in online engagement underlying perceptions of impacts.

Student, parent, and teacher accounts on internet use highlighted social media as the activity of choice with the most pervasive online use activity among adolescents and across genders. Gaming and streaming emerged also as key screen time activities for adolescents across the three narratives. However, the discussion during interviews and focus groups organically gravitated towards the discussion of social media use. Screen time impacts were conceptualized similarly across stakeholder groups, but varied in perceptions of severity and quality according to specific online activity (i.e., gaming or social media). Stakeholder groups primarily appeared to share common concerns about the perceived harms or risks (i.e., privacy and over-disclosure). However, benefits were also experienced and acknowledged (i.e., social capital). Commonalities were identified and presented, and are summarized in Table 1 along with associated perceived processes presented in Figure 1. Differences in severity and quality were also analysed below. Across stakeholders, the following key themes for impacts where identified: (i) a continuum of processes underlying impacts, (ii) stakeholder consensus on perceptions of harms, (iii) vulnerabilities predispose poor mental health, and (iv) impacts dependent on context and meaning attached. Perceptions of perceived harms (Theme 2) formed the following sub-themes: time displacement impacts, peer judgement-related impacts, sensory overload leading to hyperarousal, and context-related impacts.

### 3.2. Theme 1: A Severity Continuum of Perceived Processes Underlying Impacts

Impacts were conceptualized by students, parents, and teachers as experienced on a continuum, reflecting a broad range of positive to negative consequences with individual, social, and contextual aspects governing where an individual lies on the continuum (Figure 1). These processes were related to positive motives and outcomes powered by the “always on culture” (defined as maintaining a constant online presence powered by the symbiotic nature of social networks) [121] and relating to learning and exploration, communicating, and sharing (i.e., social evaluations, self-presentation), or for emotion regulation purposes, escapism, and control needs, further forming habits. Negative social media processes were considered when use was perceived as over-reliant for distress management or when loss of control and impaired functional consequences were experienced (for a comprehensive analysis of psychological processes underlying perceived impacts and harms, see Throuvala et al. [122]). Impacts were therefore defined by both benefits and potential perceived harms and the balance between them defined the level of normative or potentially problematic use, ranging from social facilitation and learning to compulsive use. Overall stakeholders acknowledged multiple positive processes—an opportunity for information seeking, vicarious learning and exploration, fun and enjoyment, real-time communication, peer relationship initiation and maintenance, emotional support, sharing of common interests, citizenship, self-expression, and creativity. These appeared in agreement with student conceptualisations of usage and key motivations for engagement [121].

Online communication was acknowledged as forming a major part of students’ daily communications whether at school or at home. However, the type of engagement was viewed as having significantly changed from own (parental and teacher) standards, powered by an endless stream of communication that appeared symbiotic and consistent with the “always on” culture [121]. Texting or phoning was viewed as replaced by a constant flow of chat functions:

“*We do not need to view technology as something that will destroy our lives or our children’s lives because the benefits that we see are significantly greater than the potential pitfalls*”

(I5F, 44 years, Parent).

“*I think they communicate on social media rather than ringing, texting. So the way they are communicating they are sending pictures or comments on pictures, or have threads of conversations, whether that is through Instagram or Snapchat or whatever but it is almost ‘a constant being in touch with each other’, so it is not the end of the school, ‘I will see you tomorrow’, but ‘I’ll chat with you later’*”

(I1F, 43 years, Teacher).

Participant views were largely common across groups with nuanced differences in the perceptions of the severity of the impacts. Parental accounts expressed the benefits of connectivity and how this was overshadowed by the negatives. However, parental views were also found to be polarised according to teacher perspectives:

“*A lot of mixed views so if you look at parental views, some are along the road ‘I wish you had not given it to them’ other parents on the road ‘actually I want my daughter to engage’*”

(I2F, 52 years, Teacher).

Teachers acknowledged the value of online uses for education. However, it was also experienced as partially disruptive due to difficulty to monitor how devices were used and the lack of boundaries between access for educational purposes or for recreation, in line with parental views for learning in home environments.

“*Students have the ability to research very quickly, so obviously there is a lot of time saved. I tend to direct it, give them a sheet with links for specific things I want them to look at*”

(I8F, 33 years, Teacher).

Teachers also referred to social media affordances of creating social spaces where adolescents expressed themselves and exercised autonomy, free from parental supervision:

“*So some of the girls thrive on it and love it and that is an integral part that they really enjoy, the social side of life online, they do a lot of daily communications online and if they are happy with their friendships, are comfortable with that sort of environment then great, I guess that is what I get from my role*”

(I1F, 43 years, Teacher).

“*..[the students] can use it wherever they are, without the parents knowing what they are doing. But when I was a kid, doing a bike ride, I had to say where I had been. So there is probably an element of creating their own space a little bit*”

(I8F, 33 years, Teacher).

Games, on the other hand, were viewed as offering positive outcomes overall for adolescent normative and atypical development and learning and for children faced with developmental challenges.

“*For introvert girls, gaming can be quite positive, the girls who are a bit autistic, we have used games like Minecraft, we have used the DS [Dual Screen] game, Brain Training, there is a place for that. But this is not like gaming with strangers or stuff like that. That has helped some girls who are introverts, because they don’t do social things, school can be very noisy for them and lonely, and it is hard for them getting the balance*”

(I2F, 52 years, Teacher).

However, apart from benefits, all stakeholder groups expressed negative impacts for adolescents on all domains of life—interpersonal, social, and academic. Therefore, challenges and harms formed the second theme.

### 3.3. Theme 2: Stakeholder Consensus on Perceptions of Challenges and Perceived Harms

The second theme focused specifically on consensus on impacts and harms between various stakeholder groups. These were conceptualized forming four main sub-themes: (i) time displacement impacts, (ii) peer-judgement impacts, (iii) sensory overload causing hyperarousal, and (iv) context-related impacts. Impacts were perceived as having functional (performance, task switching, and use of multiple devices), cognitive (loss or deterioration of attentional focus, and attention deficit), or emotional consequences (stress, anxiety, and obsessive-compulsive/checking behaviours) experienced both on an intrapersonal (i.e., self-esteem problems) and an interpersonal level (poor communication, lack of empathy due to asynchronous communication and lack of visual/auditory cues, egocentrism, narcissism). More specifically, perceived harms identified were primarily psycho-emotional and less safety-related (Table 1), with a small minority of teachers and parents holding extreme positions: “*Phones have replaced the booze and drugs*” (I6F, 30 years, Teacher).

*Time displacement impacts*. Excessive time spent online was a primary preoccupation amongst parents and teachers. Students admitted spending more time online than they thought or planned for, but did not share the same preoccupation with parents and teachers over the loss of time spent on social media.

“*Before I used my phone way more than I do now, until it was taken away. I still do now but I now notice how much I am using it. I had not realized how much I was using it, I thought I truly need it but I do not actually need it that much, it is a bit of a hassle when people send you stuff and get more catty about it and then you get it back and you are on the same pattern again*”

(FG1F5).

“*I literally use it excessively, like I do not go off my phone*”

(FG6F3).

Teachers perceived that time spent online displaced time spent on learning (Theme 1) and therefore, as potentially detrimental to academic achievement and counterproductive, but also at the expense of fun face-to-face engagement:

“*It is taking time from other activities that they could have been doing, the motivation for doing other things that are fun has really gone down*”

(I7M, 41 years, Teacher).

Similarly, constant stimulation from the platforms (i.e., “pull” and “push” environmental strategies [122]) was viewed as depleting students’ intrinsic resources for learning:

“*Where there is something more interactive, to the field doing some research, you can see some of them, not all of them, they are just soooo bored and it is because they are not getting that instant gratification, there is nothing really dramatic happening…So they need these intense experiences. I have to be like an entertainment system, I had to change the tasks to make them work better but still their concentration is eroding*”

(I7M, 41 years, Teacher).

Time displacement concerns raised by stakeholders related to distraction; “*They are so distracted in the classroom*” (I9F, 29 years, Teacher), either in the form of internal cues (thoughts regarding online content preoccupying an adolescent) or external triggers (receiving a notification or an alert). Concerns were expressed for the role of smartphones in distraction and the loss of focus being driven by increased social media use levels, Fear of Missing out (FoMO), nomophobia (NOMO) and online vigilance:

“*Smartphones are a cause for distraction.. I have been looking at a lot of stuff to help them with revision, helping a lot with the studying and the best thing is ‘put your phone away, disconnect, come offline, have it timetabled in. As a parent preparing for GCSEs there was a difference with their mood, taking their gadgets away, it was still vibrating and it is the FoMO, so it does cause a lot of distraction and anxiety they have FoMO*”

(I4F, 49 years, Teacher).

Parental accounts of distraction leading to loss of time from refraining from other more productive activities (i.e., homework and sports) and concurrent use while doing homework was viewed as both facilitating information search, but at the same time leading to loss of productivity. Parents believed there is a lack of knowledge and emotional readiness to block out distractions:

“*I find it irritating that I have to say to my son, ‘you have to put this down and do your homework’ and he says ‘I want to have a quick look at my messages’, so that is an issue and I suspect that is an issue for parents of girls as well*”

(I6F, 39 years, Parent).

Additionally, late sleep onset due to access to devices and poor sleep quality along with lack of physical activity and displacement of sports were primary concerns amongst adult stakeholders, but not student groups. Students instead perceived that lack of extracurricular activities led to more social media use to alleviate boredom. According to teachers, lack of sleep caused by use of devices prior or during bedtime was viewed as impacting daily performance in school. This appeared particularly difficult to control because adolescents appeared adept at deceiving parents utilising devices during bedtime.

“*Some students are really lethargic at times, and I don’t know if that is linked, probably it is linked to their phones, they’re not eating or sleeping properly*”

(I7M, 41 years, Teacher).

“*When we have meetings about achievement we ask, ‘Do you have your phone with you when you do your homework or when you go to bed?’ and a lot of the time it is because the girls are underachieving and when we ask the parents if the girls have the phone with them when they are doing homework, they have access to it and at night*”

(I4F, 49 years, Teacher).

A key parental concern with social media was the struggle to use social media in moderation, which also formed an adolescent concern. Issues relating to overuse were prominent in all accounts, acting as key drivers for adolescent engagement, such as FoMO, or NOMO, constant checking of content and overreliance on reward-seeking behaviours. Addiction was viewed as only affecting a minority of students.

*Peer judgement impacts.* These were both intrapersonal and interpersonal in nature. A major intrapersonal impact was related to anger/aggression and exposure to negative peer evaluation, social rejection or exposure to varying degrees of hateful content. Fall-outs within friendship groups were perceived to be a regular phenomenon arising from heated interactions, leading to miscommunication on chats or from lack of social skills online, resulting in inappropriate or insulting language. Teachers responded to hostile behaviours by encouraging students to reflect on such behaviours:

“*I think it is already an issue and we address it from as early as Year 7 and we had discussions...the kind of: ‘Let’s put you in that person’s position.’’ If you say something online and it is not very kind, how would you feel if that person said it to you?’ And I don’t know whether that calmed things down or not but we did have a situation, we also sent out letters to parents along those lines and explained that we had got some issues and I needed to be talking about this because there were students that were really upset about things that were happening to them*”

(I2F, 52 years, Teacher).

One teacher referred to social media as being “*an echo-chamber of emotions*” (I6F, 30 years, Teacher) which in late adolescence was perceived as also being amplified by substance and alcohol use. However, teachers reported observing backlashes when students viewed hurtful comments. In specific instances, aggressive behaviour was treated with students deleting their own accounts in response.

“*In a chat situation, you have girls who might have sent the message but thinking that is really nasty and think ‘I am really glad that is not me’….It is bullying no matter how you look at it, so there is an emotional side to it….in fact emotions drive all of it, anger wanting to be ‘leaf of the pack’ all those sorts of things. I think it is driven by emotions, like on Facebook, people put emotional comments, statuses, and obviously as the girls get older, it can be driven by alcohol. So for example, [Years] 11,12,13 is a whole different thing going on, so then they are driven by substances as well.*”

(I2F, 52 years, Teacher).

Deletion from a group chat was viewed as liberating, particularly to female adolescents.

“*I deleted my account and I am finding it great. I am going back home and I don’t have to worry about it*”

(FG1F2).

Therefore, aggressive behaviours emerged as a theme from and being displayed in various intensities, from milder to more prevalent and direct. Friendship fall-outs and aggressiveness on social media were viewed as relating to online disinhibition—the anonymity and lack of direct face-to-face communication facilitated in the online environment unleashing a negative attitude—and prompting misinterpretation. Gender-related differences (in users’ behavioural responses) rendered gender-sensitive reactions to communication online, which impacted differentially:

“*I think girls take things quite personally, so if somebody puts a picture on their wall or says something and the problem with texting is that it can be misinterpreted…there are natural problems with girls anyway, a lot of fallouts, once a girl takes a photo and puts it on Instagram, and that makes the person upset, if it’s not the flattering photo, so it’s actually like bullying, well I suppose it is a form of bullying and bullying is kind of gone outwards now, in that it is going on the internet*”

(I8F, 33 years, Teacher).

“*It is quite explicit what they send online and once a young girl masturbated for a boy online and he asked for a video, there was a lot of awareness. It was a big thing*”

(I6F, 30 years, Teacher).

Hostile behaviours were perceived as being highly prevalent also in gaming.

“*Young people playing, saying obscenities, horrible things with the characters and laughing about it—I can see a bit of that bullying over in the schools*”

(I3M, 34 years, Teacher).

An additional common concern amongst stakeholders related to self-representation on social media. Images were perceived as particularly impactful on adolescents with unhealthy and perfectionistic strives and pressure for flawlessness causing distress, low self-esteem, low mood and negatively influencing body image. Preoccupation with image curation for popularity and potential body image concerns/dissatisfaction were expressed. Additionally, an excessive emphasis on selfie-taking and the “*loss of focus on the experience in the moment*” (I7F, 49 years, Parent) was perceived as being at the expense of the experience. Parents and teachers suggested that children were overly concerned with capturing the moment rather than living it, stripping experiences from emotional investment and encouraging idealistic strives and sinister behaviours.

“*I think anxiety, depression, eating disorders, all that is a big thing and if you look at mental health and young people through the internet, online use can contribute to that to perform in a certain way, to be clever, to be beautiful, to be sinister all that*”

(I7M, 41 years, Teacher).

“*Phones and technology have the same kind of blocking effect to mental health like drugs and alcohol do. If someone is feeling anxious that might take some drugs or drink, whereas children might feel anxious and might want to take their phone. Obviously [the internet] is not so sinister like drugs and alcohol but it has the same kind of effect, a blocking effect.*”

(I9F, 29 years, Teacher).

Finally, contact risks involving risks of disclosure of personal information and exposure to pornographic or other harmful content (i.e., fake news) were discussed primarily by parents and teachers.

*Sensory overload leading to hyperarousal*. Constant connectivity was also considered as leading to social and information overload and hyperarousal by all stakeholders, but primarily by adults partially influenced by multitasking. Adolescents perceived the constant flow of information and communication, but lacked experiences to make a comparison given they have grown up in the digital era. This was viewed as forming a vicious cycle, not allowing for periods of rest or reflection on the behaviour:

“*I think there is this flood of a stimulus…When I was home, that exposure was not there…When I was young it was like don’t walk out in the dark, but now it comes to you in your home*”

(I8F, 33 years, Teacher).

“*We are the anxious ones, trying to protect them, give them time off it, just for their brain when they get in a difficult situation at school, that still happens. When we were younger, you went home, you may have told your mum. Then the next morning you still got a bit frosty, whereas now something happens everyone thinks it is their own business. They tell and then they fabricate and then someone from the school across the road says something else, and then from another school and no-one knows what is happening, and the young person does not have time to reflect on what is going on, it is just a constant thing*”

(I4F, 49 years, Teacher).

*Context-related impacts*. The most prevalent context-related impact perceived by parents and teachers was the constant checking of devices, which appeared to interfere with homework. In pursuit of instant gratification, adolescents experienced loss of sustained and uninterrupted periods of concentration and focus. The use of devices to facilitate or execute homework was a reality for many of the students. However, social media interactions were perceived as interfering with primary tasks that adolescents were unable to avoid or resist.

“*In the meetings with girls, if the phone is there in front of them and of course they cannot leave it. They have to have it because of those strikes because they need to maintain them, so many dares, to buy likes, ‘Who likes me or my picture?’ so they are afraid to lose sight. I have had to take off phones from girls at school time to put them away because they are not allowed*”

(I1F, 43 years, Teacher).

Over-reliance on online communication at the expense of offline, lack of physical activity or gradual displacement of sports or activities, and gradual loss of offline social skills were primary concerns amongst parents and carers, including sedentary lifestyle (poor diet and risk of obesity). Constant availability and access to new content and feeds, the multiplicity of social media channels, and the presence of “online audiences” were discussed by parents and teachers as increasing the social pressures placed on adolescents and leading to lack of balance. Due to the increasing accessibility and use of tablets as educational tools, boundaries between education and recreation were viewed as blurred. Another context-related impact was the rigid expectations for instant reciprocation and emotional ambivalence or experience of distress over delayed responses, difficulty in setting boundaries in online relationships and lack of confidentiality and privacy.

“*I think there is no balance, just generally. Children sleep with their phones next to them without realizing. This is one of the first things they do when they wake up so I think just in general there is a lack of balance*”

(I6F, 30 years, Teacher).

### 3.4. Theme 3: Individual Vulnerabilities Associated with Poor Mental Health

As a result of the online pressures experienced, psychosocial correlations associated with online challenges comprised three categories: first, internalizing/externalizing behaviours with mood shifts because of media feeds; second, stress, anxiety and rumination (worry over posts, preoccupation with content); and third, depressive symptoms arising from social comparison to experiences of others, self-blame and internalization of poor self-image resulting in low self-esteem and compromised self-identity. These vulnerabilities were perceived as triggered by social media and online features (i.e., “likes”) and associated with mental health.

“*Being online they ruminate a lot and they get quite low mood and girls tend to empathise and sympathise so I would say there are a lot of positive things but also a lot of negative feelings*”

(I2F, 52 years, Teacher).

“*Μy big thing is the emotional side, and what it does to their body. There is anorexia, bulimia, everything how they perceive themselves and the world…These things have affected and worsened their emotional health and well-being*”

(I9F, Teacher).

“*Actually, I think it impacts self-esteem negatively. The irony is with ‘likes’ [is that] it should boost self-esteem, but actually it has the opposite effect on it. So it brings low self-esteem. Also lots of games and apps are designed to release the endorphins. It’s like a high, so you’re potentially giving these to children, all these highs constantly, and you are matching that by continuing it. So, it is like they’re addicted to it unfortunately*”

(I4F, 49 years, Teacher).

Striving for perfection in own-generated content and images (airbrushed photos and reassurance of likes) was considered a key behaviour on social media generating unhealthy and maladaptive perfectionistic strives and pressure for flawlessness. These maladaptive cognitions were viewed as triggering anxiety or other emotional and mental health problems. Self-harm, body image concerns, and eating disorders were viewed as being on the rise and exacerbated by the use of social media, creating a vicious cycle of fall-outs. Habitual usage behaviours were also viewed as difficult to break in late adolescence:

“*I don’t think it gives poor mental-health, I think it triggers it*”

(I2F, 46 years, Parent).

“*Self-harm, a lot of copycat behaviour. It is a big problem changing this, or have a problem with the sexual orientation. You don’t know there is so much, just complete overload. They self-harm and this makes them feel better temporarily and then they feel guilty and then goes a consistent cycle*”

(I6F, 30 years, Teacher).

“*The older students still having that lifestyle of playing too long and not sleeping properly and letting it affect their relationships. That is when they get in the deep because with ‘A’ levels [advanced school qualifications] that is much harder to do*”

(I7M, 41 years, Teacher).

### 3.5. Theme 4: Impacts Dependent on Context and Meaning Attached

The fourth theme comprised impacts, which could impact adolescents’ psychological state both positively or negatively depending on the context and meaning attached, but also striking the right balance. One such example was self-expression and freedom to set boundaries:

“*It might not be wrong but it comes across this way. Its all about how It’s interpreted.*”

(FG2F2)

Self-expression was reported as a source of positive emotion for the majority of the participants, but also associated with fear concerning negative comments or negative peer evaluation. Another domain was expectancies related to online activities, which could be rewarding, but if transferred to offline contexts and life domains could lead to frustration (i.e., social acceptance online vs. acceptance into a higher institution). Increased freedom and possibilities for connection to unknown others was viewed by children as a source of opportunity to make new friends; however, it was characterized as high risk behaviour by parents or opportunities for rule breaking:

“*Read more of this and no more screen time and if you are still awake you can read a book- but she will always try to break the rules*”

(I5F, 44 years, Parent).

Interacting socially online could be a source of support and connectedness, but could also result in the experience of negative emotions from feeling left out or disconnected. Those situations and the perception/interpretation of the situation were viewed as resulting in mood shifts, elevation or deterioration of mood:

“*Now all is so much more amplified, emotionally, particularly girls, I have seen E. cry, very emotional, very upset. I mean it is a difficult age. Anyway it’s just giving them technologies, one more thing to do with alongside with the hormones*”

(I1F, 42 years, Parent).

## 4. Discussion

The present study examined stakeholders’ perceptions on impacts experienced by adolescents from their digital use. Findings corroborated challenges and harms evidenced in the literature [23,123,124]. However, the qualitative nature of the present study allowed for a comprehensive account across all three stakeholder groups and an assessment of nuanced differences in perceptions. The first theme comprised a severity continuum of processes underlying impacts, highlighting perceptions and reflections on impacts from engagement. The second theme comprised stakeholder consensus on perceptions of challenges and harms, while a third theme comprised individual vulnerabilities predisposing poor mental health. The fourth theme formed impacts which could have a positive or a negative outcome with impacts dependent on context and meaning attached for the adolescent.

There were no stark perceived differences in the conceptualization of harms across stakeholder groups (rather, differences pertained to the extent, severity and quality of those impacts). Context-related impacts were the least mentioned by adolescents given the lack of alternative perspective (i.e., life context without online engagement). Themes therefore appeared common in adolescent, parent, and teacher accounts. Parents and teachers presented a more generalized concern for the impacts and potential harms and stressed the contextual (online vs. offline balance) and time displacement aspects of adolescent online activities (i.e., psychosocial impacts) potentially due to them being the most tangible in nature and particularly given the heightened expectations during adolescence for academic achievement and its implication for occupational attainment [125]. Teachers stressed both concerns relating to displacement, but also emphasised the content-related impacts (i.e., aggression) reflecting school experiences and the increased in prevalence of cyberbullying and cybervictimization in schools [126]. Studies examining time spent on learning activities have found it to be negatively related to PSU, whereas time spent on entertainment has been associated positively with PSU and displacing time for communication unlike time spent for self-expression (i.e., gaining acceptance and image curation) [127].

The first theme comprised the conceptualization of online user experience as fluid and non-hierarchical across adolescent, parent, and teacher perspectives with positive, neutral, and negative experiences. User experience fluidity was a result of individual, social, and environmental forces as supported by the control model of engagement [122]. Therefore, data designated multiple benefits and impacts as emerging across a continuum of benefits and harms, which are context-dependent with varying degrees of quality and severity. The findings suggested that adolescents have some concern of the risks, but mostly appreciate the benefits of social media, such as social capital and self-development. Positive impacts were identified by all stakeholders and these concerned identity development and self-expression, consistent with the literature [128,129], and negative impacts depending on adolescents’ motivations for online engagement [18,59,130,131,132,133,134,135,136,137] or individual factors such as self-control and emotion-regulatory capacity [138]. Escapism for mood regulation was also highlighted as a key mechanism, consistent with previous studies. PIU on Instagram has been associated with the frequency of specific uses (i.e., watching Instagram live streaming) while on Facebook by passive use, promoting a sense of escapism from reality [139,140]. The need for escapism in adolescence has been intensified during the COVID-19 pandemic [141]. Other factors suggested as influencing the experience of harms could be personality factors, peers, parental mediation, parents’ own behaviours, and provision of alternative activities of self-worth [142,143,144,145,146,147].

The second theme referred to stakeholder agreement on the nature of the impacts. Accounts were organized in a taxonomy of time displacement, peer judgement (content), and context-related impacts reflecting cognitive, emotional, and behavioural dimensions, consistent with prior evidence [24]. Time displacement-related impacts referred to a perception of excessive time spent on social media and the level of distraction arising from use, with loss of attention on the primary task and primarily related to displacement of offline activities, which has been highly debated in the literature [16,148,149]. However, a longitudinal study conducted in the US indicated a trend towards less time spent on non-digital activities in adolescence and less in-person social interaction, suggesting displacement to online activities [2,20].

Spending more time online has been associated with problems in delivering daily activities and social interactions [25]. Distraction (an avoidance mechanism of focusing on less significant issues to avoid attending to the most crucial ones), and overstimulation were common experiences, consistent with previous findings [150]. Social overload has been found to mediate the relationship between social media use and reduced well-being levels, with FoMO moderating this relationship [151]. Establishing the relationship between time spent and psychological harms has important implications for allowing social media use in schools and home environments, and during task/homework completions as it affects an individual’s overall performance levels and well-being.

*Peer judgement-related harms* referred to aggressive and abusive communication, issues of self-representation and social rejection, cognitive rigidities associated with harms and addiction correlates, consistent with previous evidence [152]. Gaming was associated with aggressiveness and deterioration of behaviour with the use of bad language, which has been empirically investigated, while social media with misinterpretation and verbal aggressiveness were empowered by online disinhibition. Aggressiveness and cyberbullying were identified as frequent and relevant problems. This could take the form of overt action or manifest in more covert ways by means of account deletion or sext-sharing [153]. Account deletion was a reaction perhaps in an effort to better manage time [154] or as an emotional response to shield from emotional turmoil. Additionally, sexting, reported to increase significantly over the course of adolescence [155], appeared to be a risk factor for sext-sharing and therefore, incurring harm. Eco-chamber effects in social media use reported in the present study have also been reported in the literature as “digital emotional contagion” (i.e., the process of the emotional impact of peers’ emotional states on others online) via social networks [156]. Emergent experimental research suggests digital emotional contagion may vary in intensity and reflect positive or negative valence [157,158,159,160].

Self-representation problems, perfectionistic tendencies, and rigid expectations for instant reciprocation and for immediate rewards were also prevalent in stakeholders’ narratives. Research on self-representation and negative comparison has highlighted associations of social browsing on post-browsing negative affect, whereas awareness of image curation may act as a protective factor [161]. Additionally, maladaptive cognitions in the form of perfectionistic tendencies and social hopelessness have been associated with problematic social media use [162,163]. Investment in self-representation is associated with body image concerns across genders, and disordered eating with photo-based activities being particularly salient, mediated by internalization and appearance comparison [164]. Sexy self-representations on social media predisposed engagement in sexting among adolescent girls, unlike exposure to sexy self-presentations of others [165], suggesting an increased preoccupation with own and others’ constructions of online self [166].

Similarly, psychopathological symptoms and negative consequences of social media use via smartphones have been influenced by FoMO and intensity of social media use [167], whereas decreased self-esteem is linked to potentially detrimental FOMO-inspired SNS use [56]. Phubbing was another impact experienced undermining face-to-face communication, with research suggesting it is associated with problematic and addictive tendencies [61,168]. Evidence suggests that social media use may become addictive for a small minority of individuals [67,169,170]. Therefore, this harm appeared less frequently in stakeholder accounts. Mechanisms which could drive problematic social media behaviour appear to be peer pressure, poor self-regulatory mechanisms, habitual behaviour and psychosocial factors associated with it, such as fear of missing out (FOMO), social connection, reciprocal liking, and social competition [171].

Context-related impacts related to the effects of difficulty to set boundaries in online engagement or inability to find a balance between offline/online activities and patterns of use (i.e., distraction and procrastination) leading to sedentary lifestyles. Issues related to sedentariness, such as poor diet and less exercise, combined with higher frequency of accessing devices and content contribute to the development of physical and mental health problems [172,173]. Distraction and procrastination have been associated with reduced academic performance [174,175]. Rumination and expectancies for distress reduction have also been found to be positively related to the more problematic smartphone users [176].

The third theme discussed the correlates of those impacts, such as anxiety or depression or internalizing behaviours, emphasizing the scope of digital impacts and potential associations with mental health disorder symptoms. However, the direction of the relationships remains unclear [3,177], as are the strengths of the associations with suggestions for weak associations with negative outcomes [178]. FoMO and nomophobia based on their frequency and severity could trigger or act as precursors of problematic online use [179] or smartphone use [180]. Research has suggested that experiences of social rejection—intensified by youth’s narcissistic tendencies to maintain a desired self-image—were found to be associated with increased time spent and problematic social media use [166].

There is a consistent relationship across studies between cyberbullying and depression among children and adolescents [30,181]. Short sleep duration has been associated with increased digital time among adolescents [35] and social media overuse with poor sleep outcomes: (i) sleep disruption (melatonin suppression) and desynchronization of body clock–hormone imbalance and brain inflammation and lower levels of deep sleep, (ii) desensitization of the brain reward system (reward, focus and motivation), and (iii) exposure to light at night has also been associated with risk of depression [28,182,183]. The effects of excessive or problematic social media or smartphone use have been evidenced. PIU has been found to mediate the relationship between parental monitoring and low academic achievement, sleep quality, substance use, anxiety, and depression [184]. Finally, impacts were conceptualized as having the potential to be non-binary, depending on the context and meaning attached to the online interaction, consistent with research on motivations, such as self-expression, increased freedom and possibilities for exposure and relational interactions [121,185].

Limitations of the study include its small purposive non-clinical sample from one geographical area of the UK focusing on a specific developmental group, which compromises the generalizability of the findings. Exploring impacts and harms from a developmental and cross-cultural perspective or in vulnerable adolescents from youth mental health services could provide insights about universal themes and issues affecting children and adolescents. Additionally, parents of children with mental health difficulties could provide a different account about the contribution of social media in the adolescent and family life context. These insights could inform prevention programmes and media literacy curricula in an evidence-based manner, relating to the challenges adolescents encounter [186]. Second, using focus groups and in-depth interviews could also have affected the findings of the study as these were not uniform methods of analysis and may have influenced the process of qualitative research and the findings. However, the rationale in the design of the present study was for focus groups to take place and then obtain more depth and detail on the topics that arose in the adolescent focus groups and to follow-up by interviewing relevant stakeholders in order to advance the understanding on the issue [187,188]. Investigating positive outcomes was beyond the main aim of this study because the focus was specifically on the concerns and perceived consequences of internet use to inform media literacy prevention objectives. However, positive outcomes from social media use are of particular importance as the majority of the literature has tended to focus on the negative effects of media use; the many positive outcomes relating to social media use are not as often reported as such effects are less well studied [189]. Additionally, the findings may be compromised in terms of temporal validity given the rapid changes in the digital environment and the social media services offered, which may suggest a different level of interaction and generate alternative experiences and ensuing impacts for adolescents.

The present study assessed online impacts and focused on harms experienced by adolescents from a combined stakeholder perspective, providing insight and highlighting the commonalities and the differences in the perspectives. Drawing away from binary conceptualizations and embracing a spectrum perspective of psychological processes related to impacts may have important policy and prevention implications in the design of media literacy education programmes, school policies, and parental and teacher mediation. There is a need for theoretical synthesis and development of theories that account for negative impacts in normative and at-risk use, as current explanatory models focus primarily on the addictive potential of social media and technologies, but do not account for other behaviours along the continuum, such as cognitive or metacognitive impacts potentially responsible in problematic online engagement [39]. Continuum beliefs could also facilitate problem recognition and help-seeking [190] and contribute towards a more comprehensive understanding of overall engagement of adolescent screen time.

## 5. Conclusions

Research suggests that adolescents greatly benefit from the online environment, but that a minority also experience psychological challenges and harms. Understanding stakeholder conceptualizations—student, parent, and teacher—for online-related psychological harms experienced by adolescents was sought in order to develop a more coherent understanding of perceived impacts and potential harms as perceived by each stakeholder group. The findings of the present qualitative study identified positive but focused primarily on the negative impacts experienced. Adolescent online perceived challenges were conceptualised by stakeholders as running on a severity continuum from benefits to harms. There were no stark perceived differences amongst stakeholders in the conceptualization of harms; rather, differences pertained to the extent, severity and quality of social media use. Impacts were conceptualized as running on a continuum from positive to negative uses and their severity depending on the role of social media and gaming on the overall context in an adolescent’s life. Moreover, negative impacts across stakeholder groups were conceptualised as (i) time displacement impacts, (ii) peer judgement impacts (content) and (iii) context-related impacts. Concerns spanned various domains of functioning from physical (i.e., sleep), psycho-emotional (i.e., anxiety and loss of control), and cognitive (i.e., temptation-distraction), affecting both the individual and relationships on both an interpersonal and an intrapersonal level.

The findings, therefore, suggested heterogeneous challenges and potential harms—beyond e-safety and the risk of addiction—related to emotional health and depending on personal circumstances and vulnerabilities, which may contribute to poor youth mental health and well-being. Therefore, the need for emotionally healthy schools endorsing positive mindsets and addressing these adolescent challenges requires an urgent response. Stakeholder consensus about perceived harms constitute a first step in developing an understanding of the concerns, which could be embedded in harm prevention efforts and skill development in interventions.

## Figures and Tables

**Table 1:** Stakeholder perceived impacts and harms.

**Theme 1: A Severity Continuum of Perceived Processes Underlying Impacts**	
A host of positives overshadowed by the negatives	**Learning/skills acquisition** For research purposes Use as a collaborative tool Important skills for future life/Brain training **Community/Social** Social and personal development space A personal space, freedom of expression Gaming positive outcomes Benefits to introverted or vulnerable children (i.e., autistic) Major part of daily communications and social spaces Friendship maintenance and acquisition **Identity development** Threads of communication/“A constant being in touch with each other” New modes of communication rather than ringing or texting
**Theme 2:** **Stakeholder consensus on perceptions of harms**	
Time displacement-related impacts	**Excessive time spent online** **Counterproductive impacts** (academic underachievement, poor time management, distraction, compromised ability to focus, lack of deep work, procrastination, boredom/lack of activities, loss of focus on the experience) **Displacement/sedentary lifestyle** impacts (poor diets, risk of obesity, displacement of sports/activities, poor academic performance, gradual loss of offline social skills, overreliance on online communication at the expense of offline interactions, later sleep onset/sleep deprivation/poor sleep quality)
Peer judgement-related impacts	**Display of aggression/abusive communication** (explicit language, harmful/racist/hateful/violent content, cyberbullying, friendship fall-outs arising from misinterpretation of intentions and behaviours, negative influence when gaming) **Low inhibition to online disinhibition** **Expression/acceptance/rejection** (friendship fall outs, peers’ judgement on self-expression, social rejection, manipulation of peer influence and popularity, preoccupation with image curation, body image concerns/dissatisfaction, sexting, influencers/celebrities as role models, chasing flawlessness) **Addiction correlates** (preoccupation, FoMO, constant checking, rumination, loss of control, reward seeking behaviours, a “blocking effect”) **Cognitive biases/rigidities** (rigid expectations for instant reciprocation, emotional ambivalence or experience of distress over delayed responses, expectations for immediate reward/instant gratification pursuit, dependency on likes as a reward, celebrity following, black and white thinking—, i.e., account deletion as a growing backlash to hurtful comments) **Risks of disclosure** of personal information, cyberstalking, social surveillance **Risks of exposure** (pornographic, gambling-like or other harmful content (i.e., fake news, connection to unknown others, grooming/data security risks, sexting/sext-sharing, impact on sexuality, false feeling of trust) **Rumination** (emotional responses become amplified, apathy/less emotional reactivity)
Sensory overload/hyperarousal	**Social, Information and sensory overload** Constant exposure Lack of downtime/self-reflection time Amplification of insecurities Dampening of critical capacity/discernment
Context-related impacts	**Boundary-setting** (online/offline relationships, online problems carried offline, private life/disclosure confidentiality/breaching, double standards—having a separate life online, diffused boundaries between activities-gambling/gaming/social/streaming, homework/leisure, online/offline balance, digital trace long-term) **Patterns of use with perceived negative impact** (i.e., increased multitasking, habitual use, distraction)
**Theme 3: Vulnerabilities predispose poor mental health**	
For children with emotional difficulties or vulnerabilities	**Stress/anxiety/compulsive symptoms** (worry over posts, preoccupation with content) **Depressive symptoms** (social comparison or lack of similar experiences to others) **Internalizing/externalizing** (mood shifts as a consequence of browsing, self-blame, self-harm, body image concerns, reinforcing self-harm, eating disorders, internalization of poor self-image resulting in low self-esteem and compromised self-identity)
**Theme 4: Impacts dependent on meaning attached**	
Perceptions and expectancies defining impacts	**Self-expression** could be a cause for negative comments but also a source of positive emotion **Increased freedom but also possibilities for connection to unknown others** is viewed by children as a source of opportunity to make new friends, but as a source of risk exposure **Mood shifts** as a consequence of browsing could be related to elevation or deterioration of mood **Relational interactions** could cause negative emotions from feeling left out or disconnected or could be a source of support, inspiration and social capital **Instant gratification expectations** from online activities could be rewarding but damaging if transferred to offline contexts (academic, social, etc.). **Games offering positive and negative outcomes** (i.e., fun, social spaces, hand-eye coordination) but when displacing learning then deemed negative

## Data Availability

The data of the present study is part of a larger qualitative study exploring media use and recommendations for prevention. Parts of this larger study have not yet been published and data will not be made available until after the final papers have been published.
